# Thermal performance of brick and stone masonry: Cumulative heat flux dataset for main orientations and under diverse seasonal conditions

**DOI:** 10.1016/j.dib.2020.106599

**Published:** 2020-12-01

**Authors:** Loukas Georgiou, Christiana Panteli, Angeliki Kylili, Paris A. Fokaides

**Affiliations:** a Frederick Research Center, Cyprus; b Frederick University, School of Engineering, Cyprus

**Keywords:** Numerical simulation, FEM, Thermal performance, Building element, Heat flux

## Abstract

This dataset consists of the hourly heat flux for four seasons and orientations of 15 different construction configurations of brick and stone masonry combined with insulation system solutions. The analysis was conducted with the use of Finite Element Modelling (FEM). The development of the models and the investigation of their thermal performance was conducted with the use of thermal modelling and numerical simulation analysis with COMSOL Multiphysics. For this purpose, a transient 2D multi- dimensional, time- dependent simulation model on finite elements was developed. The governing equations of heat transfer were considered as well as the convection and radiation heat transfer coefficients in accordance to the ISO 6946:2017 [1].

## Specifications Table

**Table:** 

Subject	Energy Engineering, Building Physics
Specific subject area	Heat transfer, Heat flux, Finite Elements Modeling (FEM), Masonry, Insulation
Type of data	Tables; Figures
How data were acquired	Solar radiation tool (PVGIS) [2] for boundary conditions Finite elements modelling tool (COMSOL Multiphysics) for heat flux
Data format	Processed data
Parameters for data collection	Data collection for the development of the numerical simulation model was performed using experimental building elements. Geometric parameters and thermophysical properties were obtained experimentally. Data collection for the development of the temperatures profiles, imposed on the surfaces of the masonry, were obtained with the use of PVGIS.
Description of data collection	The thermophysical properties of the brick and stone masonry were defined using experimental methods. In particular, their thermal properties have been measured based on the analysis of the temperature response of each masonry test specimen to heat flow impulses with the use of a measuring instrument for direct measurement of heat transfer properties. The exterior boundary data for the numerical models was defined using climatic conditions data extracted from the PVGIS tool for the calendar months January (winter), April (spring), July (summer), and October (autumn) and for four orientations − azimuth 0°, 90°, 180° and 270° and using the sol-air temperature equation. The heat flux data was extracted from the FEM tool, based on the numerical simulation studies performed.
Data source location	Nicosia, Cyprus, 35.18° N, 33.37°E
Data accessibility	http://dx.doi.org/10.17632/xkjbtgdyys.1

## Value of the Data

• The data provided in this work may be used as a guideline for building element configurations to improve the overall thermal and energy performance of buildings.
• These data can be used for other scientific studies that are correlating brick and stone masonry configurations with insulation system solutions for the improvement of building energy performance.
• The data set will support researchers to correlate the heat losses resulting from heat transfer in various building construction configurations.
• By providing the data of the energy performance of widely- used building elements in the construction sector, researchers can exploit the annual total loads to develop novel energy optimisation approaches.

## 1 Data Description

**Fig. 1:**
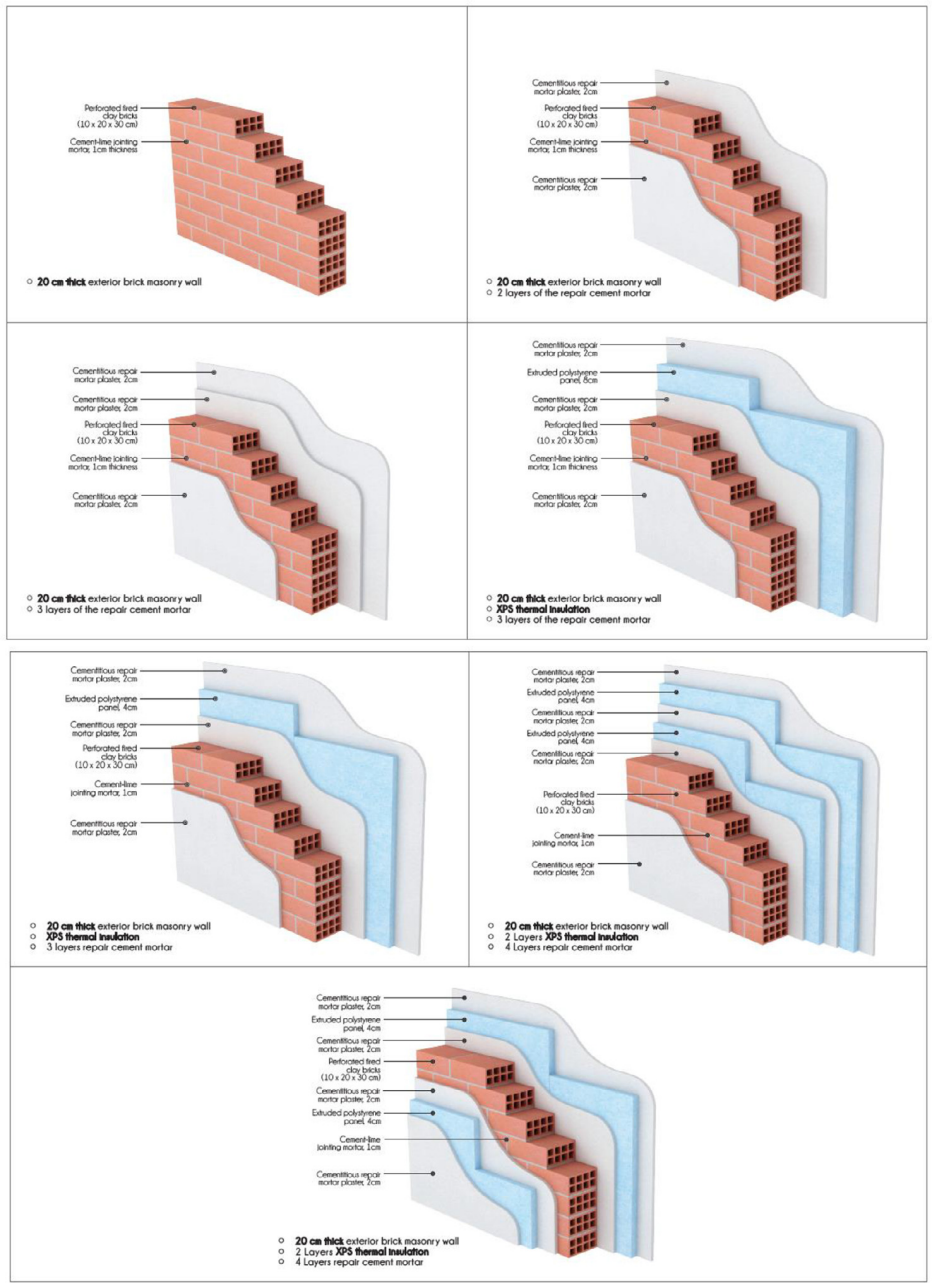
Investigated building elements: Brick walls.

**Fig. 2:**
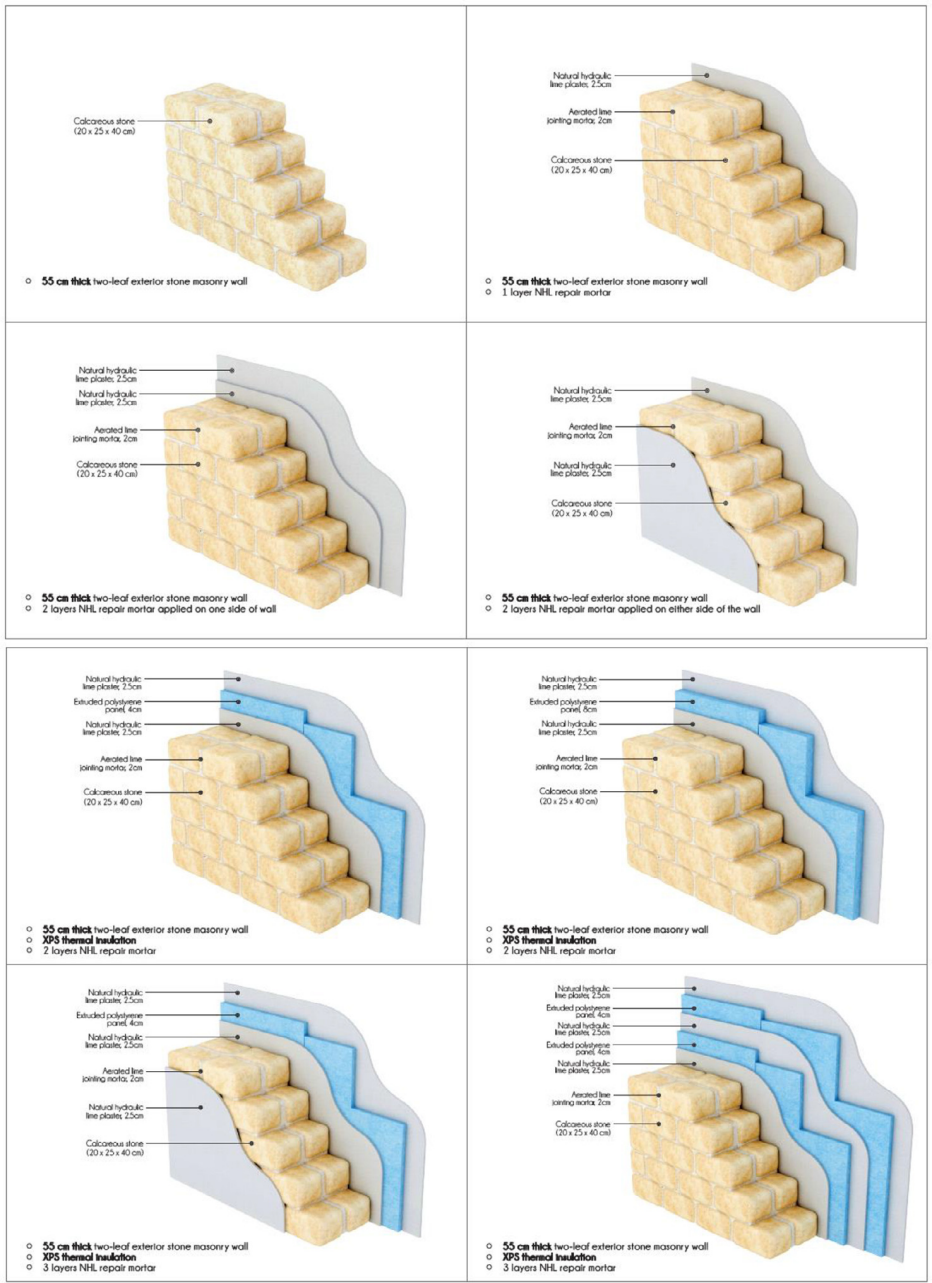
Investigated building elements: Stone walls.

In Figs. 1 and 2, the building elements which were investigated in terms of this work are provided. The investigated configurations are described in detail in Table 5, under the Experimental Design, Materials and Methods section. The dataset is comprised of 60 Tables of hourly cumulative heat flux [W/m²] (Table 1 – 60) data acquired through the implementation of a numerical simulation study for each construction case, each calendar month and each orientation. For the same dataset, 60 Figures of total heat flux magnitude [W/m²] and 60 figures of temperature boundary conditions [C°] are presented. The summary of findings are presented by 8 Figures representing the cumulative heat flux values for all investigated months and cumulative heat flux by season for each orientation.

**Fig. 3:**
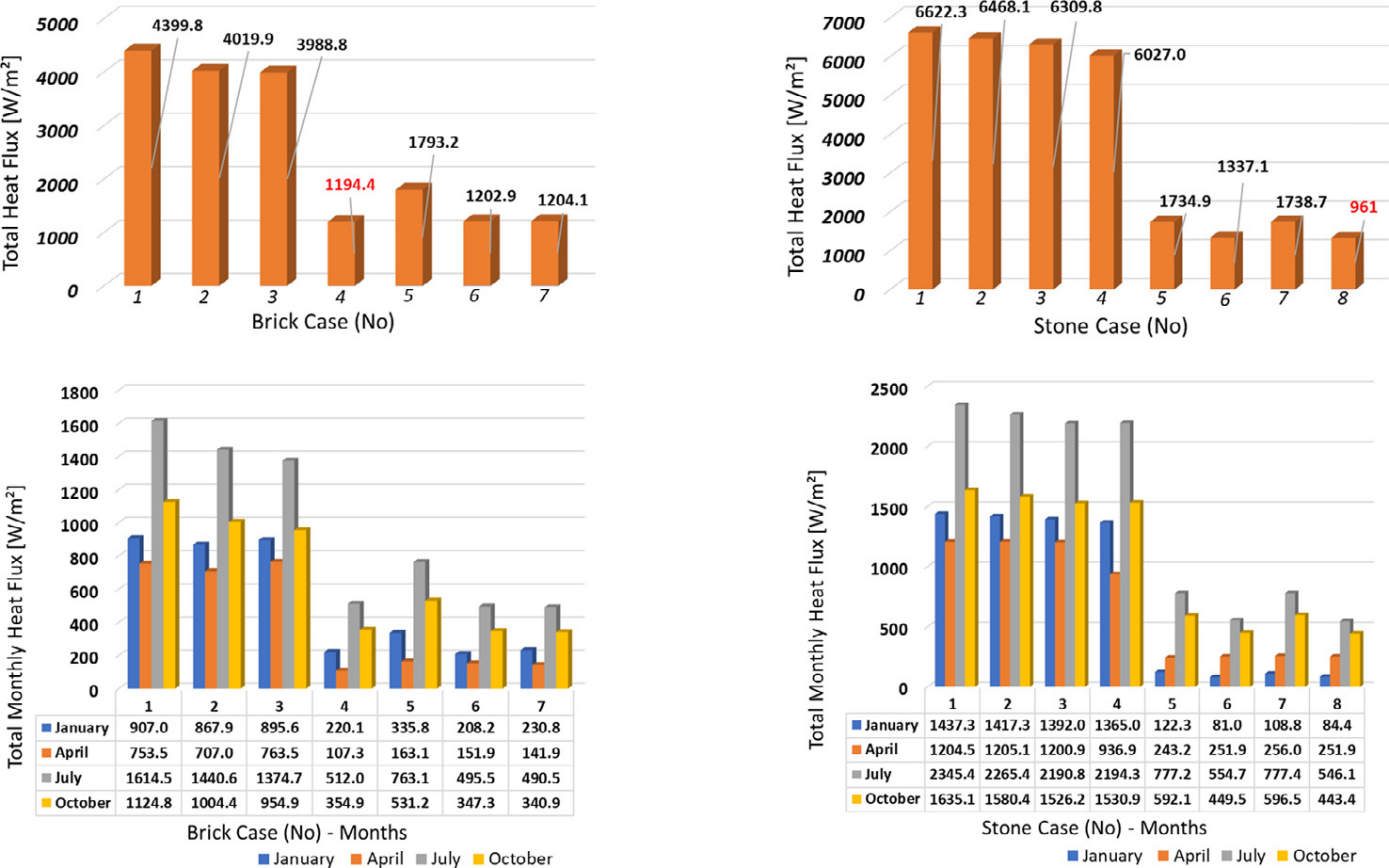
Cumulative heat flux and monthly heat flux values for the months under investigation – Brick walls (left), Stone walls (right).

The results of this work have been summarized and are presented in this section. Fig. 3 illustrates the cumulative heat flux values and the monthly cumulative heat flux values for the months under study for the brick and stone masonries. Summary tables of cumulative

heat flux by calendar month, building element and orientation are also provided below (Table 1, Table 2, Table 3, Table 4). The heat flux values are highlighted in red- orange- green colour, where red indicates the highest values and green indicates the lowest values.

**Table 1:** Cumulative heat flux by calendar month for azimuth 0°.

**Table 2:** Cumulative heat flux by calendar month for azimuth 90°.

**Table 3:** Cumulative heat flux by calendar month for azimuth 180°.

**Table 4:** Cumulative heat flux by calendar month for azimuth 270°.

**Table 5:** Hourly. Investigated configurations.

Case	Construction Description
B1	20 cm thick exterior brick masonry wall without thermal insulation or coating (reference case)
B2	20 cm thick exterior brick masonry wall without thermal insulation, coated with 2 layers the repair cement mortar hereby developed
B3	20 cm thick exterior brick masonry wall without thermal insulation, coated with 3 layers of the repair cement mortar hereby developed
B4	20 cm thick exterior brick masonry wall with XPS thermal insulation and 3 layers of the repair cement mortar hereby developed
B5	20 cm thick exterior brick masonry wall with XPS thermal insulation and 3 layers of the repair cement mortar hereby developed
B6	20 cm thick exterior brick masonry wall with two layers of XPS insulation installed on one side and 4 layers of the repair cement mortar hereby developed
B7	20 cm thick exterior brick masonry wall with two layers of XPS insulation installed on either side and 4 layers of the repair cement mortar hereby developed
S1	55 cm thick two-leaf exterior stone masonry wall without thermal insulation or coating (reference case)
S2	55 cm thick two-leaf exterior stone masonry wall without thermal insulation and 1 layer of the NHL repair mortar hereby developed
S3	55 cm thick two-leaf exterior stone masonry wall without thermal insulation and 2 layers of the NHL repair mortar hereby developed applied on one side of the wall
S4	55 cm thick two-leaf exterior stone masonry wall without thermal insulation and 2 layers of the NHL repair mortar hereby developed applied on either side of the wall
S5	55 cm thick two-leaf exterior stone masonry wall with XPS thermal insulation and 2 layers of the NHL repair mortar hereby developed
S6	55 cm thick two-leaf exterior stone masonry wall with XPS thermal insulation and 2 layers of the NHL repair mortar hereby developed
S7	55 cm thick two-leaf exterior stone masonry wall with XPS thermal insulation and 3 layers of the NHL repair mortar hereby developed
S8	55 cm thick two-leaf exterior stone masonry wall with XPS thermal insulation and 3 layers of the NHL repair mortar hereby developed

*B: Brick Case: S: Stone Case.

The Supplementary Material contains the transient 2D numerical simulation models, developed in Comsol Multiphysics, which delivered the results provided in this work. The numerical simulation models include all the input information and values used for their development. For each configuration, the geometry and the thermophysical properties of the materials (density, thermal conductivity, heat capacity, diffusion coefficient) are defined. The heat transfer tab contains the equations used for the time- dependent study and provides detailed information for the physical model, the dependent variables, the heat transfer mechanisms and the thermodynamics. Furthermore, the initial values, the thermal insulation and internal and external temperatures/ temperature profiles are also provided in this tab. The mesh settings, element size and element size parameters used for each simulation study are also defined. The numerical simulation models also provide information of the study settings, including the input for physics and variables selection, values of dependent variables, as well as the mesh selection solver configurations such as absolute tolerance and time stepping. The results tab of the simulation models also contain the heat flux tables, which are presented in this work.

## 2 Experimental Design, Materials and Methods

A transient 2D numerical model based on finite elements was developed with the use of a FEM tool (Comsol Multiphysics 5.0). The parameter based on which the alternative building elements were evaluated was the total heat flux. Information on the examined meshes, the domains, the calculation physics as well as the boundary conditions is provided below, while the specific input information and values used can be found in the numerical simulation models, found in the Supplementary Material:

Concerning the physics of the finite element analysis, the fundamental law governing all heat transfer is the first law of thermodynamics, commonly referred to as the principle of conservation of energy.

$$\rho C_{p}\left(\frac{\partial T}{\partial t}+\left(u\;\cdot \nabla \right)T\right)=\;--\left(\nabla \cdot q\right)+\tau :S\;--\frac{T}{\rho }\;\frac{\partial \rho }{\partial T}{|}_{\rho }\left(\frac{\partial \rho }{\partial t}+\left(u\;\cdot \nabla \right)\rho \right)\;+\;Q$$

The assumptions made for simplification of the solved equation are the following:

• Heat transfer interfaces use Fourier's law of heat conduction
• Mass conservation, ie density and the velocity are related through equation
  $$q_{i}\;=\;--k\frac{\partial T}{\partial x_{i}}$$
• Viscous heating and pressure work negligible
• Heat transfer in solids (velocity = 0)

Based on the above, the equation solved in each cell is the following:

$$\frac{\partial \rho }{\partial t}+\nabla \cdot \left(\rho v\right)=\;0$$

Concerning the computational domain, two-dimensional rectangular multi- layer geometries, simulating 15 different construction alternatives of brick and rock masonries were developed. Triangular meshing was employed, of which the maximum and minimum element size were 0.0064 and 1.28E-5 meter, respectively. The maximum element growth rate was 1.1, and the curvature factor 0.2 with the resolution of narrow regions being 1. The preset for the mesh quality was chosen to be extremely fine, with the purpose of the accuracy of the results being directly proportional to the mesh size.

The boundary conditions were set in the form of a temperature profile for the external surface of the wall and constant temperature for the internal surface of the wall. Regarding the exterior wall boundary conditions, the use of the sol-air temperature was employed. Sol-air temperature T_(sol-air) is defined as

$$T_{\mathrm{sol}-\mathrm{air}}=T_{O}+\frac{\left(\alpha I-\Delta Q_{\mathrm{ir}}\right)}{h_{O}}$$

The analysis was conducted for four calendar months, delivering representative results for the entire calendar year (January – Winter, April – Spring, July – Summer, October – Autumn), as well as for the four main orientations (azimuths 0°, 90°, 180°, and 270°). The ambient temperature values and the total solar irradiation for Nicosia, Cyprus, were retrieved from the PVGIS [2] tool of JRC, making use of also two reanalysis-based solar radiation datasets, namely ECMWF [3] ERA-5 and COSMO-REA [4]. Post- processing of the Tsol-air values was conducted, to define the most representative day of the month, to be used for the analysis. For the overall heat transfer coefficient $h_{o}$, the convection and radiation heat transfer coefficients were considered according to the EN standard 6946:2007 [1]. The heat exchange with the sky dome was neglected. Regarding interior boundary conditions, the internal temperature was assumed to be 20 °C.

## Ethics Statement

No ethical issues are associated with this work.

## Transparency Document. Supporting Information

Transparency data associated with this article can be found in the online version at https://data.mendeley.com/datasets/xkjbtgdyys/2<https://nam03.safelinks.protection.outlook.com/?url=https%3A%2F%2Fdata.mendeley.com%2Fdatasets%2Fxkjbtgdyys%2F2&data=04%7C01%7Cr.nayal%40elsevier.com%7Cd8a354da47784882aa6608d8955f6db6%7C9274ee3f94254109a27f9fb15c10675d%7C0%7C0%7C637423586364649163%7CUnknown%7CTWFpbGZsb3d8eyJWIjoiMC4wLjAwMDAiLCJQIjoiV2luMzIiLCJBTiI6Ik1haWwiLCJXVCI6Mn0%3D%7C1000&sdata=j%2B2Qn9XErhe4W8c0VjuL6KjF2SLgeKIoK8uRe9yj%2BhQ%3D&reserved=0

## Declaration of Competing Interest

The authors declare that they have no known competing financial interests or personal relationships whichhave, or could be perceived to have,influenced the work reported in this article.
